# Supplementary figures and images for: Rab11 suppresses neuronal stress signaling by localizing dual leucine zipper kinase to axon terminals for protein turnover
Source: eLife. 2024 Oct 30;13:RP96592. doi: 10.7554/eLife.96592 (PMC11524585; doi:10.7554/eLife.96592)

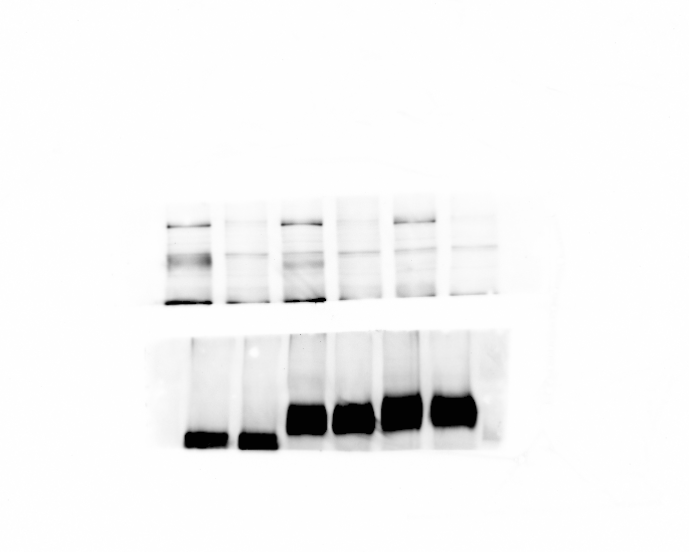

Supplement: Figure 2—source data 1. [file elife-96592-fig2-data1.zip › Figure 2-source data 1/Figure 2D_Biotin + GFP.tif]

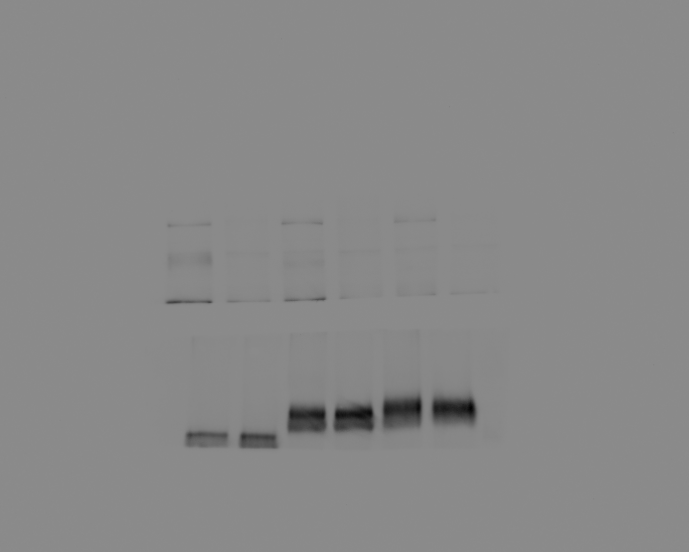

Supplement: Figure 2—source data 1. [file elife-96592-fig2-data1.zip › Figure 2-source data 1/Figure 2D_GFP_Unsaturated.tif]

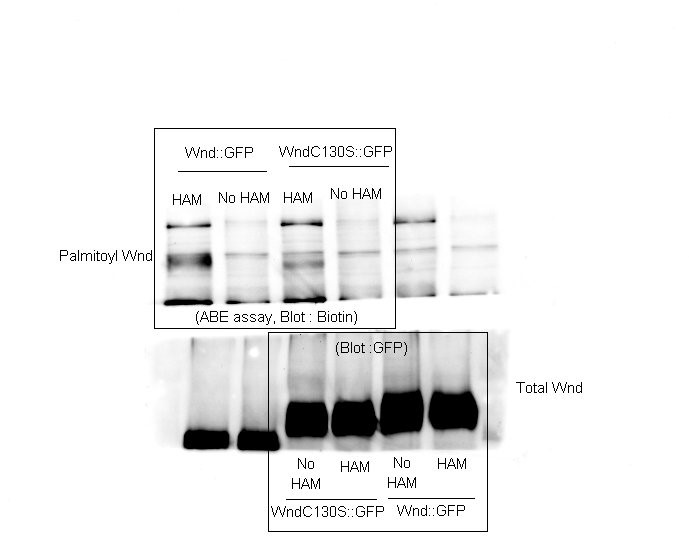

Supplement: Figure 2—source data 2. [file elife-96592-fig2-data2.zip › Figure 2-source data 2/Figure 2D_Biotin + GFP_lablled.jpeg]

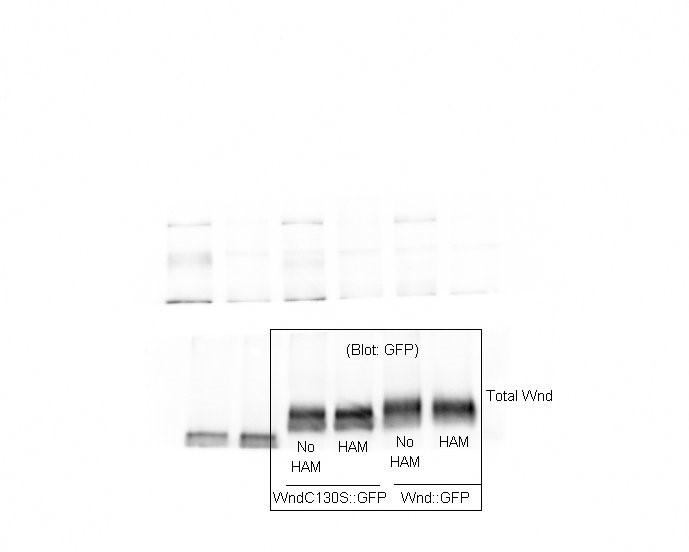

Supplement: Figure 2—source data 2. [file elife-96592-fig2-data2.zip › Figure 2-source data 2/Figure 2D_GFP_Unsaturated_lablled.jpeg]

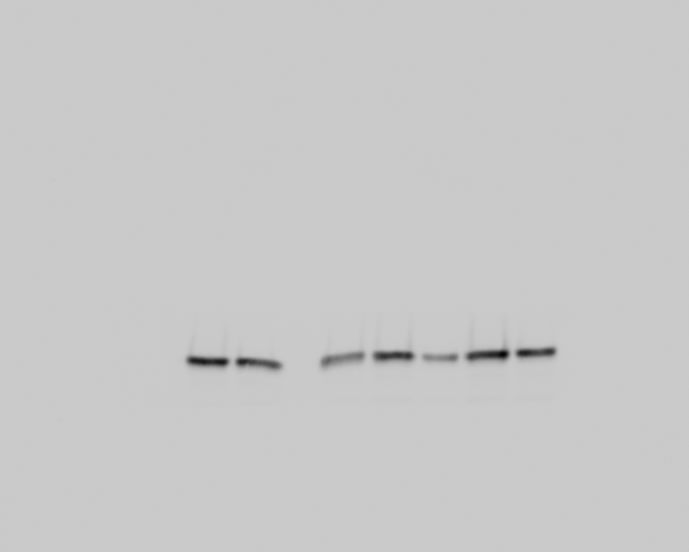

Supplement: Figure 3—source data 1. [file elife-96592-fig3-data1.zip › Figure 3-source data 1/Figure 3A_Elav_sample#1.tif]

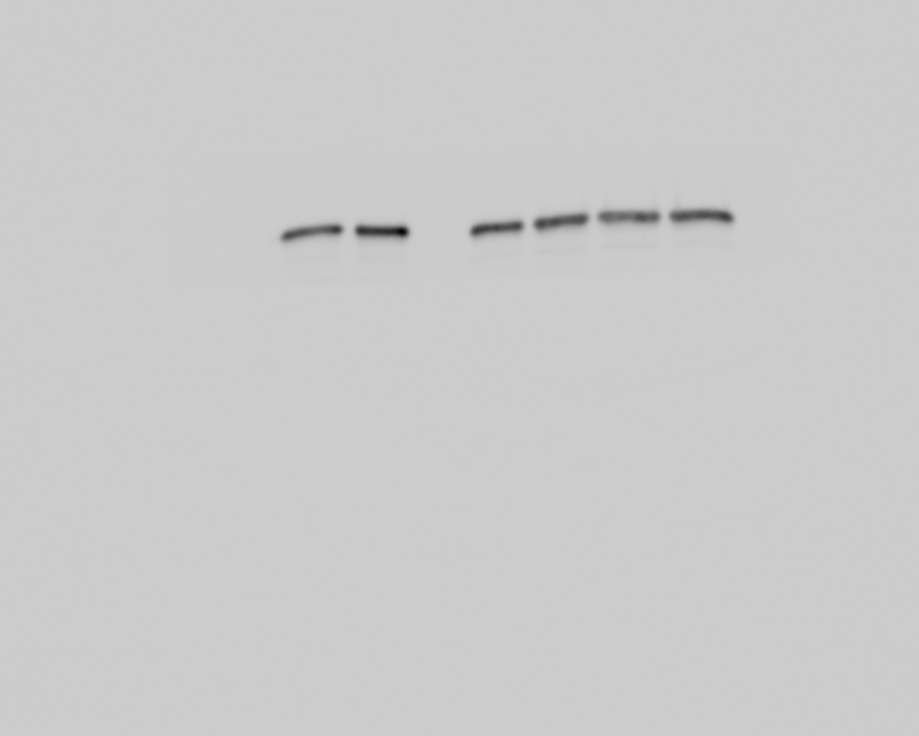

Supplement: Figure 3—source data 1. [file elife-96592-fig3-data1.zip › Figure 3-source data 1/Figure 3A_Elav_sample#2-4.tif]

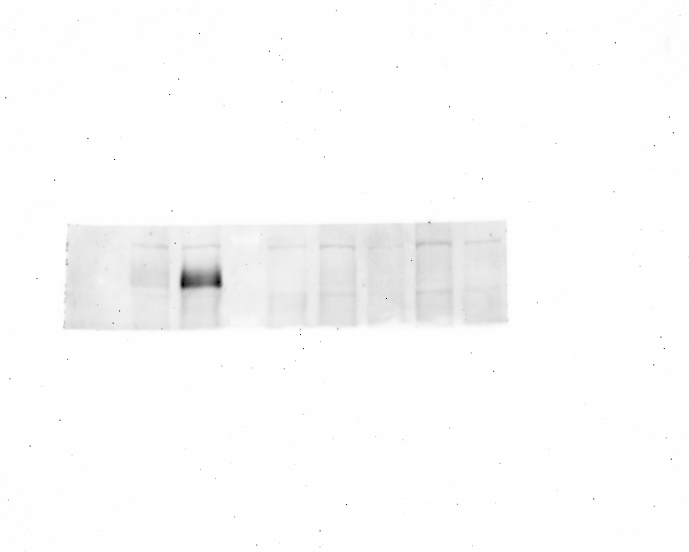

Supplement: Figure 3—source data 1. [file elife-96592-fig3-data1.zip › Figure 3-source data 1/Figure 3A_GFP_sample #1.tif]

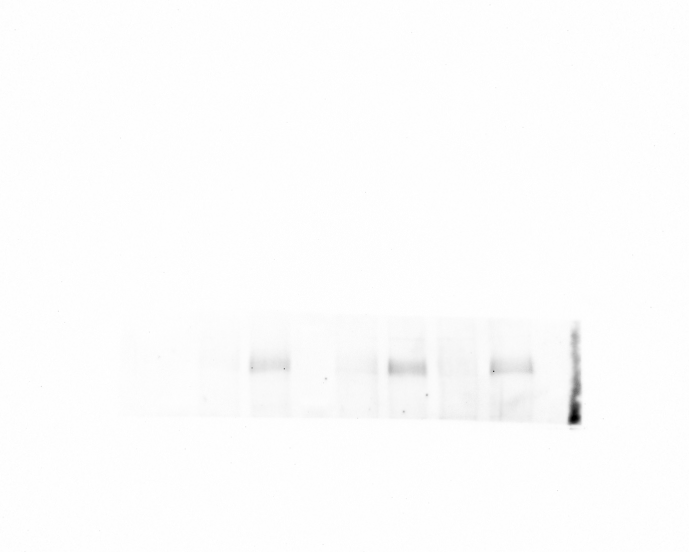

Supplement: Figure 3—source data 1. [file elife-96592-fig3-data1.zip › Figure 3-source data 1/Figure 3A_GFP_sample #2-4.tif]

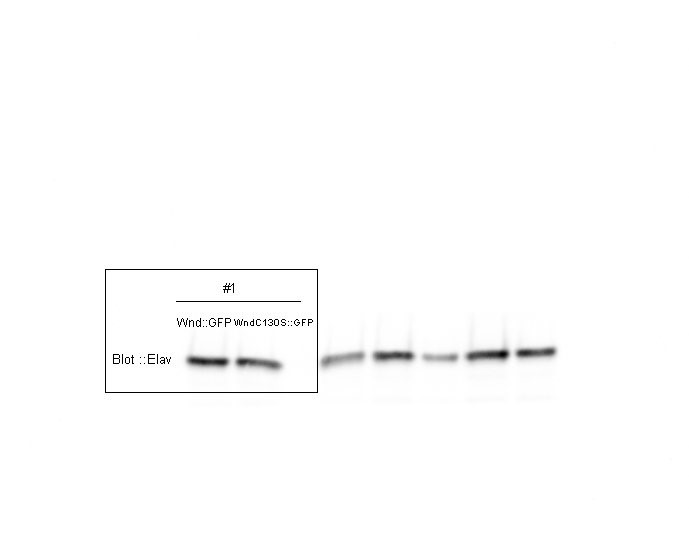

Supplement: Figure 3—source data 2. [file elife-96592-fig3-data2.zip › Figure 3-source data 2/Figure 3A_Elav_sample#1_lablled.jpeg]

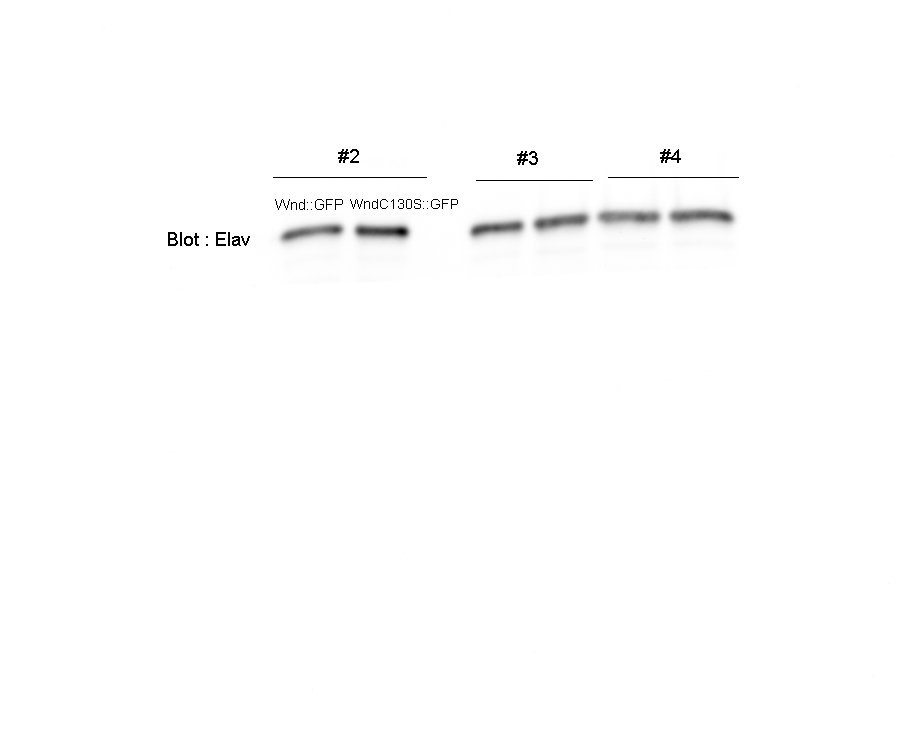

Supplement: Figure 3—source data 2. [file elife-96592-fig3-data2.zip › Figure 3-source data 2/Figure 3A_Elav_sample#2-4_lablled.jpeg]

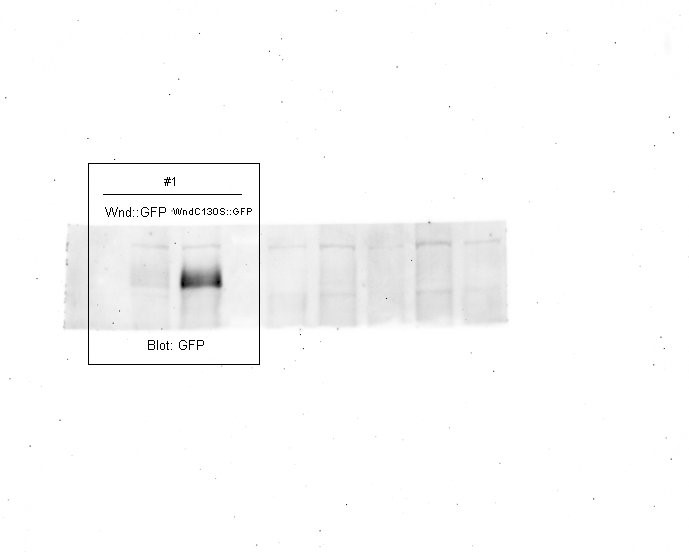

Supplement: Figure 3—source data 2. [file elife-96592-fig3-data2.zip › Figure 3-source data 2/Figure 3A_GFP_sample #1_lablled.jpeg]

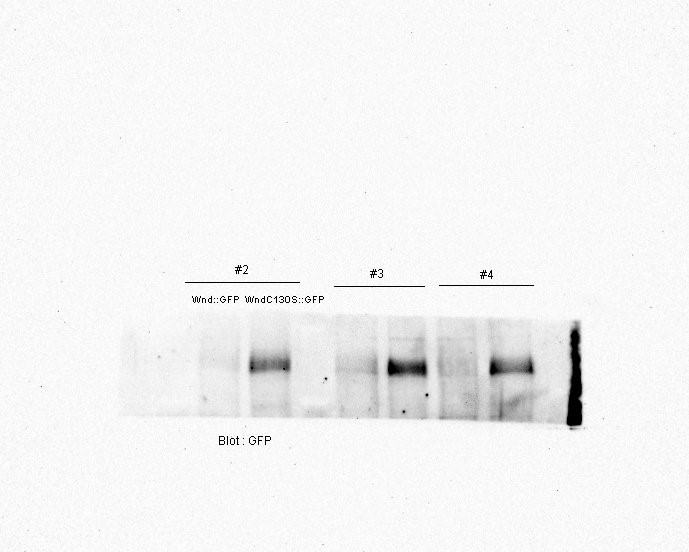

Supplement: Figure 3—source data 2. [file elife-96592-fig3-data2.zip › Figure 3-source data 2/Figure 3A_GFP_sample #2-4_lablled.jpeg]

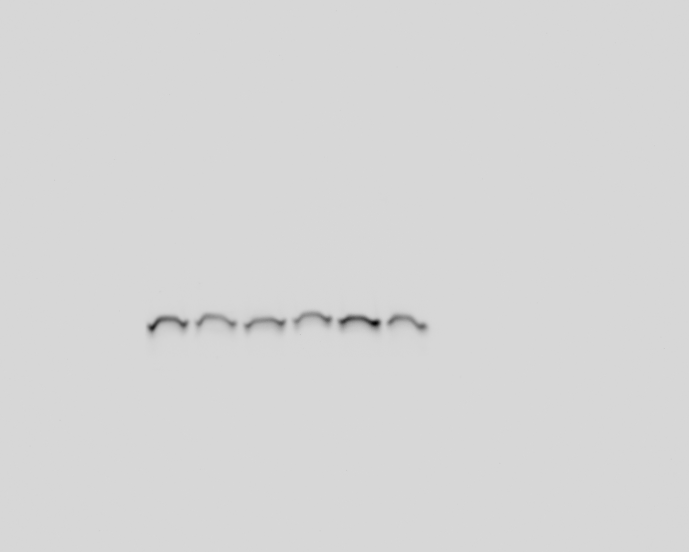

Supplement: Figure 6—source data 1. [file elife-96592-fig6-data1.zip › Figure 6-source data 1/Figure 6E_Elav_sample #1-3.tif]

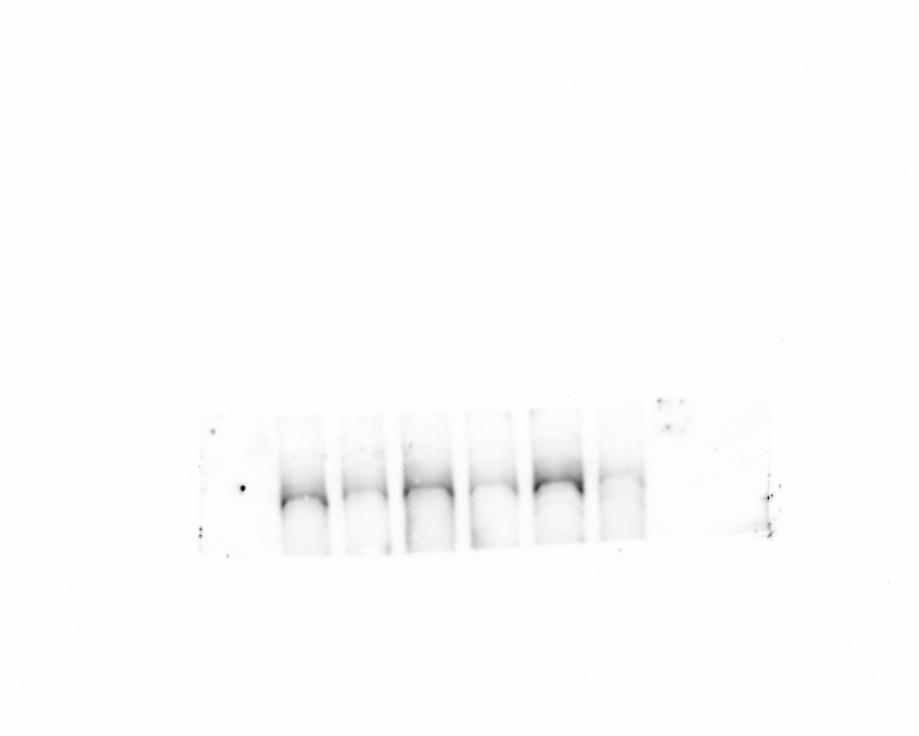

Supplement: Figure 6—source data 1. [file elife-96592-fig6-data1.zip › Figure 6-source data 1/Figure 6E_Wnd_sample #1-3.tif]

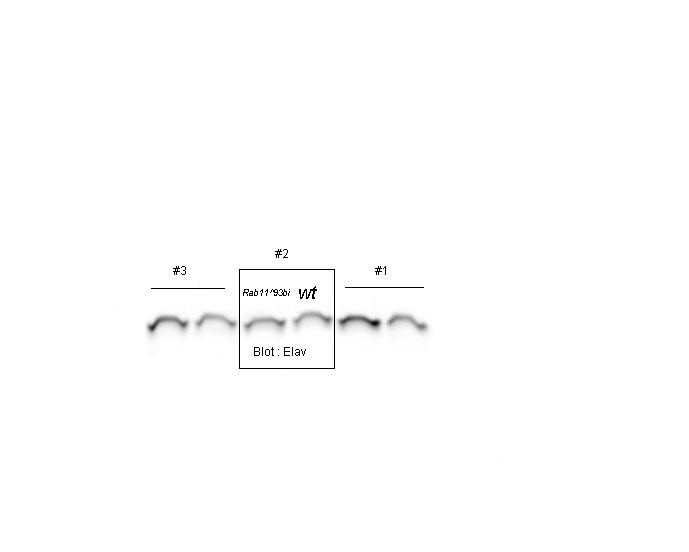

Supplement: Figure 6—source data 2. [file elife-96592-fig6-data2.zip › Figure 6-source data 2/Figure 6E_Elav_sample #1-3_lablled.jpeg]

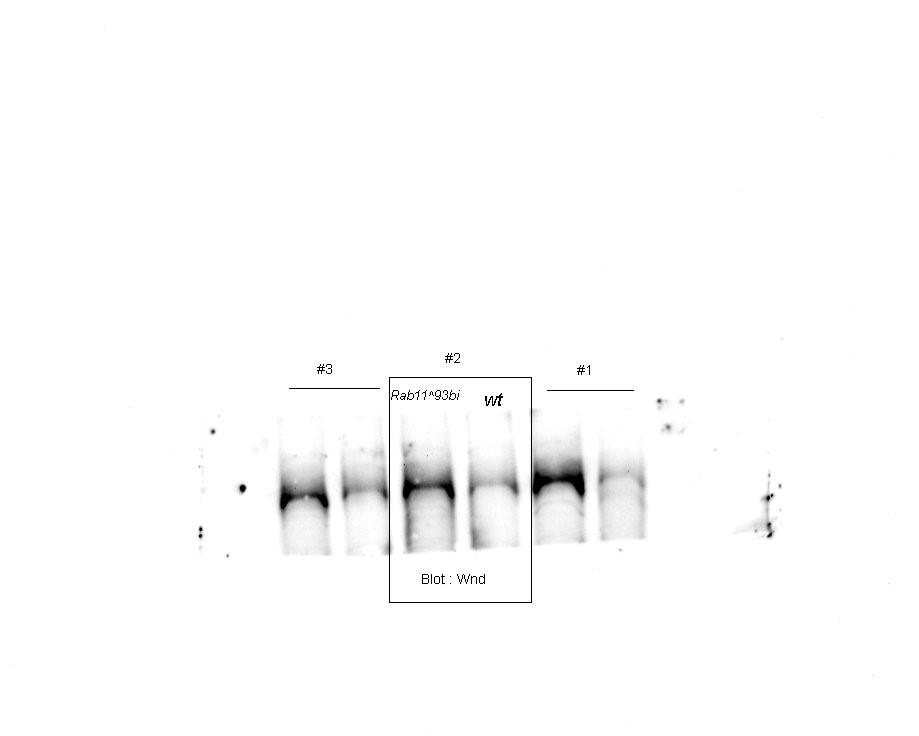

Supplement: Figure 6—source data 2. [file elife-96592-fig6-data2.zip › Figure 6-source data 2/Figure 6E_Wnd_sample #1-3_lablled.jpeg]
